# Culture Conversion Among HIV Co-Infected Multidrug-Resistant Tuberculosis Patients in Tugela Ferry, South Africa

**DOI:** 10.1371/journal.pone.0015841

**Published:** 2011-01-06

**Authors:** James C. M. Brust, Melissa Lygizos, Krisda Chaiyachati, Michelle Scott, Theo L. van der Merwe, Anthony P. Moll, Xuan Li, Marian Loveday, Sheila A. Bamber, Umesh G. Lalloo, Gerald H. Friedland, N. Sarita Shah, Neel R. Gandhi

**Affiliations:** 1 Department of Medicine, Montefiore Medical Center and Albert Einstein College of Medicine, Bronx, New York, United States of America; 2 Tugela Ferry Care and Research Collaboration (TF CARES), Tugela Ferry, South Africa; 3 University of Michigan School of Medicine, Ann Arbor, Michigan, United States of America; 4 Harvard University School of Medicine, Boston, Massachusetts, United States of America; 5 Philanjalo, Tugela Ferry, South Africa; 6 Health Systems Research Unit, Medical Research Council, Cape Town, South Africa; 7 Nelson R. Mandela School of Medicine, University of KwaZulu-Natal, Durban, South Africa; 8 Yale University School of Medicine, New Haven, Connecticut, United States of America; 9 Department of Epidemiology and Population Health, Montefiore Medical Center and Albert Einstein College of Medicine, Bronx, New York, United States of America; McGill University, Canada

## Abstract

**Background:**

Little is known about the time to sputum culture conversion in MDR-TB patients co-infected with HIV, although such patients have, historically, had poor outcomes. We describe culture conversion rates among MDR-TB patients with and without HIV-co-infection in a TB-endemic, high-HIV prevalent, resource-limited setting.

**Methods:**

Patients with culture-proven MDR-TB were treated with a standardized second-line regimen. Sputum cultures were taken monthly and conversion was defined as two negative cultures taken at least one month apart. Time-to-conversion was measured from the day of initiation of MDR-TB therapy. Subjects with HIV received antiretroviral therapy (ART) regardless of CD4 count.

**Results:**

Among 45 MDR-TB patients, 36 (80%) were HIV-co-infected. Overall, 40 (89%) of the 45 patients culture-converted within the first six months and there was no difference in the proportion who converted based on HIV status. Median time-to-conversion was 62 days (IQR 48-111). Among the five patients who did not culture convert, three died, one was transferred to another facility, and one refused further treatment before completing 6 months of therapy. Thus, no patients remained persistently culture-positive at 6 months of therapy.

**Conclusions:**

With concurrent second-line TB and ART medications, MDR-TB/HIV co-infected patients can achieve culture conversion rates and times similar to those reported from HIV-negative patients worldwide. Future studies are needed to examine whether similar cure rates are achieved at the end of MDR-TB treatment and to determine the optimal use and timing of ART in the setting of MDR-TB treatment.

## Introduction

Multidrug-resistant tuberculosis (MDR-TB, resistance to at least isoniazid and rifampin) is an increasingly important global threat [1], with an estimated 440,000 cases worldwide in 2008 [2]. Compared with drug-susceptible TB, MDR-TB is associated with poorer treatment outcomes because the medications are less potent, cause greater adverse events, and must be taken for a minimum of 18–24 months. Although the MDR-TB epidemic was initially focused in Eastern Europe, growing evidence shows that reported cases of MDR-TB in sub-Saharan Africa are rising rapidly, raising concerns for a catastrophic convergence with the HIV epidemic [3], [4], [5].

Cure rates in MDR-TB/HIV co-infected patients have historically been lower than those in HIV-negative patients, in large part due to higher mortality [6], [7]. Most studies, however, were conducted before the availability of antiretroviral therapy (ART). The addition of ART to MDR-TB treatment may improve survival and cure rates for MDR-TB/HIV co-infected patients.

Although cure of MDR-TB requires treatment with second-line TB drugs for a minimum of 18-24 months, conversion of sputum culture from positive to negative has become a valuable, intermediate outcome [8], [9]. A Latvian study of MDR-TB demonstrated that subjects who culture converted within 2 months of treatment initiation were more likely to achieve cure than those who did not [10], and other studies have demonstrated that culture conversion is associated with lower rates of relapse and failure [8]. Although smear microscopy may be more rapid and less expensive than culture, its use as an intermediate outcome is severely limited, especially in patients with HIV co-infection, who are more likely to have paucibacillary disease [11]. As a result, culture conversion is now being used as an interim outcome for predicting treatment success in MDR-TB [12]. Few data, however, are available regarding culture conversion in MDR-TB and HIV co-infection in the ART era.

South Africa has experienced a dramatic rise in MDR-TB cases over the past decade. In KwaZulu-Natal (KZN) province, the MDR-TB caseload increased 10-fold between 2001 and 2007 to a MDR-TB prevalence of 30 cases per 100,000 population [13], [14], [15]. The HIV co-infection rate among MDR-TB cases is approximately 70% [15]. To address the high MDR-TB prevalence and rates of HIV co-infection, we established an integrated community-based treatment program to provide concurrent MDR-TB and HIV treatment in Tugela Ferry, South Africa. In this study, we sought to describe culture conversion rates among the first cohort of HIV co-infected MDR-TB patients treated concurrently with MDR-TB and antiretroviral therapy in our program.

## Methods

This is a retrospective, observational study of consecutive patients initiating MDR-TB treatment between Feburary 1, 2008 and February 28, 2009. Patients were eligible if they lived in Tugela Ferry district, had culture-confirmed MDR-TB, and started second-line TB treatment. Patients were excluded if they had resistance to amikacin, kanamycin, capreomycin or any fluoroquinolone.

### Ethics Statement

This study was approved by the institutional review boards at Albert Einstein College of Medicine, Yale University, the University of KwaZulu-Natal, and by the KwaZulu-Natal Department of Health. The data used in this study were collected as part of routine medical care into the hospital clinical chart. As these were simply for clinical care, patients were not asked to give informed consent at the time of these clinical encounters. For the purposes of this retrospective study, the requirement for informed consent was waived by the ethics committees listed above, as all data had been previously collected during the course of routine medical care and did not pose any additional risks to the patients.

### Setting and treatment program

Tugela Ferry is a resource-poor, rural area in KwaZulu-Natal province, South Africa, with a TB incidence of 1,100 per 100,000 population and MDR-TB incidence of 100 cases per 100,000 in 2007 [15]. More than 80% of MDR-TB cases were HIV co-infected.

Patients presenting with signs and symptoms of TB were evaluated with sputum microscopy, culture and drug-susceptibility testing (DST), as previously described [16]. Those suspected of having TB were empirically started on standard first-line TB therapy while awaiting culture results. Upon culture-confirmation, MDR-TB patients were treated with a standardized regimen of kanamycin, ofloxacin, ethionamide, ethambutol, pyrazinamide, and cycloserine (intensive phase) for 4 months following culture conversion (defined as two consecutive negative cultures at least one month apart) and a minimum of 6 months. Patients were continued on this regimen, without kanamycin, for an additional 18 months (continuation phase). TB drugs were dosed by weight and modified in response to severe adverse effects. Third-line TB drugs and surgical treatment were not routinely used. Extensively drug-resistant (XDR) TB cases were referred to the TB referral hospital in Durban [17] and were not eligible for this study.

All patients were offered HIV testing and HIV co-infected patients initiated ART (stavudine, lamivudine, and either efavirenz or nevirapine) as soon as they were tolerating MDR-TB therapy, regardless of CD4 cell count.

Following a brief hospital admission for MDR-TB treatment initiation, patients received treatment as outpatients by modified directly-observed therapy. Nurses and community health workers visited patients' homes daily to administer both MDR-TB and ART medications during the intensive and continuation phases, respectively. Patients were seen monthly by a physician in the MDR-TB/HIV clinic. Sputum culture and drug susceptibility testing was performed monthly and CD4 count and HIV viral load were assessed every 6 months. All treatment was free of charge and patients were reimbursed for travel expenses.

### Outcomes and analysis

The primary outcome was the proportion of MDR-TB patients who culture converted. Secondary outcomes included 1) time to culture conversion, 2) stratified analysis by HIV status, and 3) mortality within the first 6 months of MDR TB therapy.

Culture conversion was defined as two consecutive negative sputum cultures at least one month apart. Timing of culture conversion was categorized as occurring: (1) on first-line therapy, before initiating second-line TB drugs (SLDs), (2) within 2 months of SLD initiation (early conversion), and (3) within 6 months of SLD initiation. Time-to-conversion was also analyzed using product limit estimates and Kaplan-Meier curves, where the time-to-conversion was calculated as the number of days since initiating second-line TB drugs. Median values were compared using the Wilcoxon rank-sum test. Patients who culture-converted on first-line therapy were excluded from this time-to-conversion analysis.

To identify clinical and demographic predictors of conversion on first-line TB therapy and early culture conversion, we used a Cox proportional hazards regression model for the following variables: age, gender, prior TB treatment, sputum smear status, HIV-status, BMI, baseline hemoglobin, CD4 count, HIV viral load, and ART use prior to starting MDR-TB therapy.

## Results

Forty-five culture-confirmed MDR-TB patients initiated treatment and met eligibility criteria for this study (table 1). Of these, 30 (67%) were women, with a median age of 33 years (IQR 27-38), and 30 (67%) were sputum smear-positive. Eleven (24%) patients were resistant only to isoniazid (INH) and rifampin (RIF); 33 (73%) had resistance to INH, RIF, and streptomycin (SM); and one (2%) had resistance to INH, RIF, SM and ethambutol. Only 16 (36%) patients had previously received TB treatment and only 3 (7%) had previously been treated for MDR-TB with second-line TB drugs.

**Table 1:** Baseline characteristics by HIV status.

Characteristic	All subjects	HIV-neg	HIV-pos	p-value
	(n = 45)	(n = 9)	(n = 36)	
Female – n (%)	30 (65)	6 (67)	24 (67)	1.0
Median Age (IQR)	33 (27–38)	28 (20–38)	34 (29–37)	0.26
Median BMI (kg/m^2^), median (IQR)	18.8 (15.8–20.3)	20.5 (18.9–22.6)	17 (15.7–19.5)	0.025
Hemoglobin (g/dL), median (IQR)	10.6 (9.2–12.4)	11.6 (10.3–14.6)	10.4 (8.6–12.3)	0.083
HIV-positive – n (%)	36 (80)	--	--	--
Median CD4 count at baseline, cells/mm^3^ (IQR)	--	--	189 (85-265)	--
On ART at start of MDR-TB treatment – n (%)	--	--	21 (60)	--
Smear-positive – n (%)	30 (67)	7 (78)	23 (64)	0.70
Any previous TB – n (%)	16 (35.6)	1 (11)	15 (42)	0.13
Previous treatment for MDR-TB – n (%)	3 (6.7)	0 (0)	3 (8.3)	1.0
Mean number of sputum samples sent in first 6 months (SD)	5 (4–6)	7 (5–7)	5 (4–6)	0.18
Median number of days from initial sputum collection to initation of MDR-TB therapy (IQR)	74 (44–106)	90 (54–113)	69 (44–103)	0.67

IQR: interquartile range; MDR-TB: multidrug-resistant tuberculosis; ART: antiretroviral therapy;

BMI: body mass index.

Thirty-six patients (80%) were HIV co-infected and of these, 21 (60%) were already receiving ART when MDR-TB treatment was initiated. Eleven patients initiated ART after starting MDR-TB treatment (median 57 days, range 22–116). The remaining four co-infected patients did not initiate ART within the first six months of treatment (two died, one was transferred to another facility and one had a CD4 count of 935). At MDR-TB treatment initiation, the median CD4 cell count was 189 cells/mm^3^ (IQR: 85-265) and 58% had viral loads that were undetectable (<400 copies/mL; among those with baseline viral load available [n = 12]). HIV-infected patients had a lower median BMI than HIV-uninfected patients (17 vs. 20.5 kg/m^2^, p = 0.03). There were no other significant differences in baseline characteristics between HIV co-infected and HIV-negative patients.

### Culture Conversion

Prior to starting MDR-TB therapy, 11% (4/36) of HIV co-infected patients and 33% (3/9) of HIV-negative patients (p = 0.13) converted their sputum culture to negative on empiric first-line TB treatment. One patient was transferred immediately after the start of therapy and did not have sufficient culture data to evaluate conversion. Four additional patients had no culture within 30 days of MDR-TB treatment initiation. Although all four of these patients remained culture-negative for the duration of follow-up, the time of conversion could not be identified and these patients were excluded from further analysis.

Of the remaining 33 patients with a positive culture at the start of MDR-TB therapy, 29 (88%) achieved culture conversion within the first 6 months of treatment. These included 23 of 27 (85%) MDR-TB/HIV co-infected and 6 of 6 (100%) HIV-negative patients (p = 1.0). Of the four HIV co-infected patients who did not culture convert, three died, and one refused further treatment within the first six months of treatment. Thus, no patients remained persistently culture-positive on treatment.

**Figure 1:**
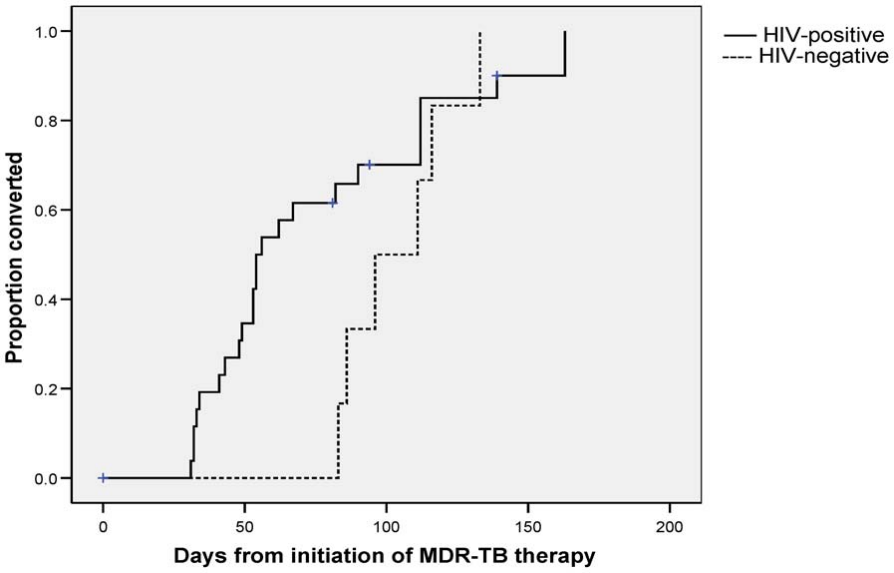
Kaplan-Meier plot of time to sputum culture conversion, by HIV-status.

The median time to culture conversion on second-line therapy was 62 days (IQR 48-111). The median time-to-conversion for HIV co-infected patients was 54 days (IQR 41-90), whereas for HIV-negative patients it was 103.5 days (IQR 86-116; Figure 1). No other patient demographics or clinical characteristics were associated with early sputum conversion (less than 60 days; data not shown).

Overall, of the 45 subjects included in this study, five (11%) were transferred to another facility, one (2%) refused further treatment, and four (9%) died while receiving therapy. The four patients who died were all HIV-positive, and death occurred after a median of 116 days in treatment for MDR-TB (range 1–199 days). As of July 1, 2009, 35 patients (78%) were alive and in follow-up having been on treatment for a mean of 333 days (SD 112).

## Discussion

We examined culture conversion rates among HIV co-infected and HIV-negative MDR-TB patients receiving treatment in a rural area of South Africa. Our findings were very favorable, demonstrating that nearly all patients achieved culture conversion within the first six months, irrespective of HIV status. Moreover, the culture conversion time for HIV co-infected patients was similar to reports of HIV-negative patients in the published literature. These early data provide optimism that a strategy of concurrently treating MDR TB and HIV may lead to favorable outcomes for co-infected patients.

Culture conversion is generally the first goal of MDR-TB therapy and is used as an early endpoint in MDR-TB studies [12]. Although most of the data supporting use of this endpoint are from studies in drug-susceptible TB, Holtz et al. [10] demonstrated a patient-level relationship between shorter time-to-culture conversion and final treatment outcome. Others have found a similar association between culture conversion rates and treatment success [8], [9]. Most published cohorts of MDR-TB treatment, however, have had few, if any, HIV co-infected subjects, and the majority of those that did are from the 1990s, predating the availability of combination ART [18], [19]. As a result, there are few data available from the ART-era comparing culture conversion rates and time-to-culture conversion between HIV co-infected and HIV-negative patients [20].

In our study, we found culture conversion rates to be similar between the two groups. No patients in either group remained persistently culture-positive (i.e., in jeopardy of becoming a treatment failure). Although a few patients in the HIV group did not meet official criteria for culture conversion, this was due to the fact that each suffered a competing outcome (patient either died, or defaulted) before completing six months of MDR therapy. The median time to culture conversion in HIV co-infected subjects was also similar to those of HIV-negative patients published in the literature worldwide [8], [10], [21], and comparable to the HIV-negative subjects in our cohort. Our high rates of culture conversion add to those recently reported among MDR-TB/HIV co-infected patients in Lesotho [22], although that group had higher mortality rates. It will be important to follow these patients' cultures throughout the continuation phase to be sure that they do not revert to positive after discontinuation of the injectable agent.

An important limitation of this study is that of survival bias. Because of the long time required for TB culture and DST, patients in this study survived a median 74 days from initial sputum collection to start of appropriate MDR-TB treatment. As we have previously shown, this delay is associated with extremely high mortality rates and many patients never receive appropriate therapy [23]. Nonetheless, the objective of this study was to show MDR-TB/HIV co-infected patients' response to concurrent therapy. Thus, although a sizeable proportion of co-infected patients may have died prior to receiving therapy, our study is valuable because it demonstrates that those who do receive therapy are likely to achieve culture conversion.

Although studies from the 1990s, before the availability of ART, suggested substantially worse treatment outcomes for HIV co-infected MDR-TB patients compared to HIV-negative patients [24], our early findings provide hope that a strategy of integrating second-line TB therapy and ART will improve MDR-TB cure rates in HIV co-infected patients. Even though our sample size was limited and final 24-month treatment outcomes are needed to definitively demonstrate the effectiveness of this strategy, our high rates of culture conversion and lower mortality rate pave the way for improved cure rates in MDR-TB and HIV co-infection, even in rural, resource-limited settings. Mired by the dual epidemics of TB and HIV, South Africa now faces a rising tide of MDR-TB/HIV co-infection and these data should provide welcome news for the concurrent treatment of both diseases.
